# Genome-wide association study identifies candidate genes contributing to flowering time variation in *Lotus japonicus* in Japan

**DOI:** 10.5511/plantbiotechnology.24.1023a

**Published:** 2025-03-25

**Authors:** Tomomi Wakabayashi, Stig U. Andersen, Sachiko Tanaka, Shusei Sato, Masayoshi Kawaguchi, Ko Kato, Hiroaki Setoguchi

**Affiliations:** 1 Division of Biological Science, Graduate School of Science and Technology, Nara Institute of Science and Technology, 8916-5, Takayama-cho, Ikoma, Nara 630-0192, Japan; 2 Graduate School of Human and Environmental Studies, Kyoto University, Yoshida-nihonmatsucho, Sakyo-ku, Kyoto, Kyoto 606-8501, Japan; 3 Collaborative Organization for Research in Women’s Education of Science, Technology, Engineering, and Mathematics, Nara Women’s University, Kitauoya Higashi-machi, Nara, Nara 630-8285, Japan; 4 Department of Molecular Biology and Genetics, Aarhus University, Universitetsbyen 81, DK-8000 Aarhus, Denmark; 5 National Institute for Basic Biology, Nishigonaka 38, Myodaiji, Okazaki, Aichi 444-8585, Japan; 6 Graduate School of Life Sciences, Tohoku University, 2-1-1, Katahira, Aoba-ku, Sendai, Miyagi 980-8577, Japan; 7 Center for Digital Green-innovation, Nara Institute of Science and Technology, 8916-5, Takayama-cho, Ikoma, Nara 630-0192, Japan

**Keywords:** flowering time, genome-wide association study, *Lotus japonicus*, wild accessions

## Abstract

Flowering time is an important factor in plant fitness and local adaptation. Genome-wide association (GWA) studies have allowed the identification of candidate genes in certain plant species for various traits, including flowering time. *Lotus japonicus* is widely found throughout the Japanese archipelago. To obtain flowering time data with more prominent difference as more suitable indicator of environmental adaptation, flowering time data were collected for 132 wild accessions originating from various points across this region under shorter day length conditions than in previous studies. The results showed latitudinal variations in flowering time, with southern accessions flowering earlier. Comparing data from four flowering times with varying conditions revealed greater differences under a shorter day length. It is likely that day length significantly affects flowering time in this species. GWA analyses were conducted on flowering time variation measured in this study and the ratios between flowering time under different conditions. Candidate genes different from previous study were detected, including orthologues of known flowering time genes in each analysis. Correlation tests between flowering time and strongly detected single-nucleotide polymorphisms (SNPs) in the GWA analysis suggested that approximately 60% of flowering time variation can be explained by the two main SNPs. This result suggests that the majority of the variation could be explained by a small number of genetic factors. Considering the strong association with flowering time variation, these candidates may be responsible for these differences and therefore can be related to local adaptation in this species.

## Introduction

Germination and flowering are two of the four stages of plant life cycles, and the timing of these two phases is dictated by environmental factors. Flowering time is particularly critical for wild plants, since it is directly linked to fitness through reproductive success (Schemske et al. 1978). A large number of wild plant species have intraspecific flowering time variations, many of which are related to local adaptation, and several studies have focused on flowering time as a factor in species adaptation (Dittmar et al. 2014; Hall and Willis 2006; Keller et al. 2012; Leinonen et al. 2013).

Improvements in sequencing technology have enabled the identification of genes of many species that are associated with various phenotypes using genome-wide association (GWA) studies. GWA studies use a large number of shared alleles, can detect genetic factors that regulate natural variation among wild populations, including adaptive phenotypic differences, and have been used to identify genes related to diverse phenotypes, such as in *Arabidopsis thaliana* (Atwell et al. 2010), rice (Zhao et al. 2011), soybean (Zhang et al. 2015), and *Lotus japonicus* (Shah et al. 2020). While previous GWA studies have focused on agricultural varieties, recent research has attempted to identify adaptive loci with GWA using high-density SNPs in the conserved alleles of wild accessions (Atwell et al. 2010; Fournier-Level et al. 2011; Mustamin et al. 2023; Shah et al. 2020; Yu et al. 2016). Some of these studies were directed toward possible key candidate genes related to flowering time, as this is an important trait for plant adaptation and reproductive success (Burgarella et al. 2016; Sasaki et al. 2015; Shah et al. 2020).

The Japanese archipelago extends across a large latitudinal range (approximately 20 degrees), which includes numerous distinct environments. For example, the day length differs between the most northern and southern points by up to 4 h, and other environmental factors, such as temperature and precipitation, vary greatly throughout the archipelago in relation to its mountainous topology and prevailing westerly and seasonal winds. *L. japonicus* is a legume model plant that is distributed across the entire Japanese archipelago and wild accessions from natural populations collected in this country have exposed (Hashiguchi et al. 2012) genome-wide polymorphisms. Previous studies have shown that this species has intraspecific flowering time variation (Kai et al. 2010; Kawaguchi 2000; Shah et al. 2020), and given its presence in various environments in Japan, it is conceivable that *L. japonicus* can adapt to a range of habitats. By combining genetic information and flowering time differentiation in these wild accessions, we can identify the genetic factors associated with local adaptation.

Genes responsible for flowering time have been detected in genetic and physiological studies of several model plants, including *Arabidopsis*, *Populus* sp., and *Glycine max*. Flowering time is controlled by four major genetic pathways (temperature, photoperiod, autonomous, and gibberellin pathways), containing more than 100 genes (Srikanth and Schmid 2011), and temperature and photoperiod are integral for the determination of flowering time in wild environments (Henderson and Dean 2004). Most of these pathways are shared among angiosperms, while some consist of different cascades and/or genes between phyletic groups. Leguminosae is a family composed of a large number of species and includes many useful plants. Common flowering time pathways and genes have been revealed in several legume species, and the *E* series genes have been previously acknowledged, particularly in soybean; candidate genes have also been detected in a common bean study (Raggi et al. 2019; Tsubokura et al. 2014). However, there are few studies that do not depend on *A. thaliana* homologous genes with known flowering time. The determination of specific flowering time pathways and genes in legumes requires association studies with flowering time traits and genome-wide polymorphisms.

GWA analyses for flowering time and other traits of *L. japonicus* have been performed on phenotype data collected in greenhouses with 16-h light period and constant temperature of 20°C in Aarhus, Denmark, and in a field in Osaki, Miyagi, Japan (38°27′36″ N, 141°05′24″ E) in 2017 and 2018, and several candidate genes were revealed in the latter (Shah et al. 2020). In 2017 and 2018 at Miyagi, the seeds were sown on March 30 and April 6, respectively, and transplanted to the field on April 28 and May 8, respectively. The day length during this period was about 14 to 15 h, and the temperature was about 0 to 30°C. However, because the flowering time is determined by external factors, the phenotype varies depending on the cultivation conditions. Moreover, this species is widely distributed in areas with shorter day length than the photoperiods used in previous studies, and *L. japonicus* is a long-day plant, therefore, it is expected that there will be more accessions that are less likely to induce flowering under shorter-day conditions than under longer-day conditions. Accordingly, it is considered that the phenotypic differences within the species will become more prominent, and that we would be able to obtain data that is more suitable as an indicator of environmental adaptation. In this study, to evaluate the flowering time under short-day conditions as an indicator of environmental adaptation and compare responses to different day lengths, we gathered flowering time data in a short-day condition in Okazaki, Aichi, Japan (34°57′ N, 137°09′ E) since southern accessions prosper under this factor in wild populations. At this location, the day length on the winter solstice is approximately 9 h 50 min, and on the summer solstice it is approximately 14 and a half hours, making it possible to grow the plant under short-day conditions under natural light conditions. In addition, we conducted GWA analyses for flowering time variation and ratios between Aichi and the results from Miyagi in 2017 and 2018 obtained in Shah et al. 2020. Comparing flowering time of common accessions between these different environments could return difference of critical day length among accessions, and GWA analysis for the ratios could detect candidate genes related to the difference.

Flowering time variations are generally a gradual trait and *L. japonicus* is not an exception, although few studies have focused on the combination of effects of multiple genetic factors. Along with GWA analysis, we conducted correlation tests between flowering time variation and the genotypes of the main SNPs detected, considering a combination of genetic factors to estimate the degree of variation attributable to the SNP genotypes detected by GWA analysis. GWA analysis is an “SNP by SNP” method, and therefore adopting these analyses together would be effective for revealing the mechanisms of maintenance of continuous flowering time polymorphism in this species. Moreover, our results would contribute to the understanding of local adaptation through *L. japonicus* flowering time.

## Materials and methods

### Plant material and measurement of flowering time

**Figure 1:**
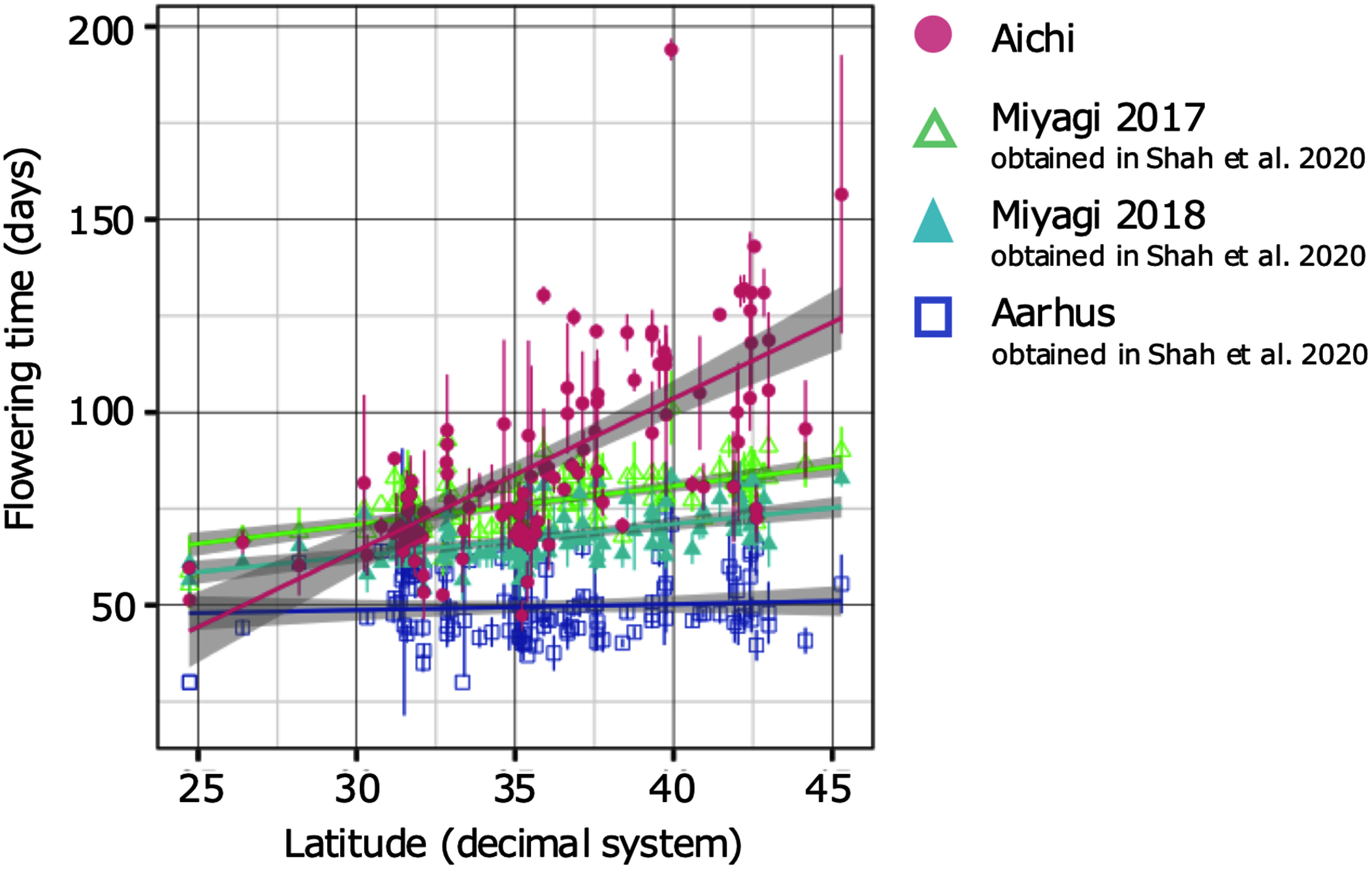
Figure 1. Variations in flowering time of Japanese wild accessions under four conditions across latitudes. Flowering time is shown on the vertical axis (days), and latitude where accessions originated are shown on the horizontal axis. Bars denote standard deviations of flowering days in each accession. Each color indicates flowering time measurement conditions of greenhouses at Aichi, Aarhus, and a field at Miyagi in 2017 and 2018, in pink, deep blue, light green, and light blue, respectively. The flowering time data at Aarhus and Miyagi were obtained in Shah et al. (2020). Each line and gray area represents the regression line and the 95% confidence interval, respectively.

The flowering time phenotypes of 132 wild Japanese *L. japonicus* accessions (Figure 1 and Supplementary Table S1) were collected. Seeds from 100 and 28 accessions were provided by NBRP (Legume Base) and Aarhus University, respectively. In addition, four accessions (Tomamae, Hakkoda, Kitsunezaki, and Tokunoshima) previously collected and cultivated after two generations of inbreeding by self-pollination were utilized.

Before sowing, the seed coat was partially removed with sandpaper; 15 seeds from each accession were sown on 1.0% agar in a 90 mm diameter, 15 mm deep Petri dish, and germinated in a growth chamber (Biotron LPH410S; NK Systems, Osaka, Japan) under a 16 h day : 8 h night cycle at 25°C for 1 week. Individuals from each accession were planted in three polyethylene pots 9.0 cm in diameter and 20 cm in height filled with potting compost, expanded vermiculite, and soil. The potted plants were placed in a greenhouse (Okazaki, Aichi, 34°57′ N, 137°09′ E) on January 31, 2014 and were grown under natural light conditions of approximately 10.5–14.5 h of daylight during the experimental period, since *L. japonicus* behaves as a long-day plant in the wild. To avoid temperature fluctuation effects, we set to a constant 25°C in the greenhouse. After 1 week, the plants were thinned, leaving only the largest individual in the pot. At this stage, each plant had three to five compound leaves.

**Figure 2:**
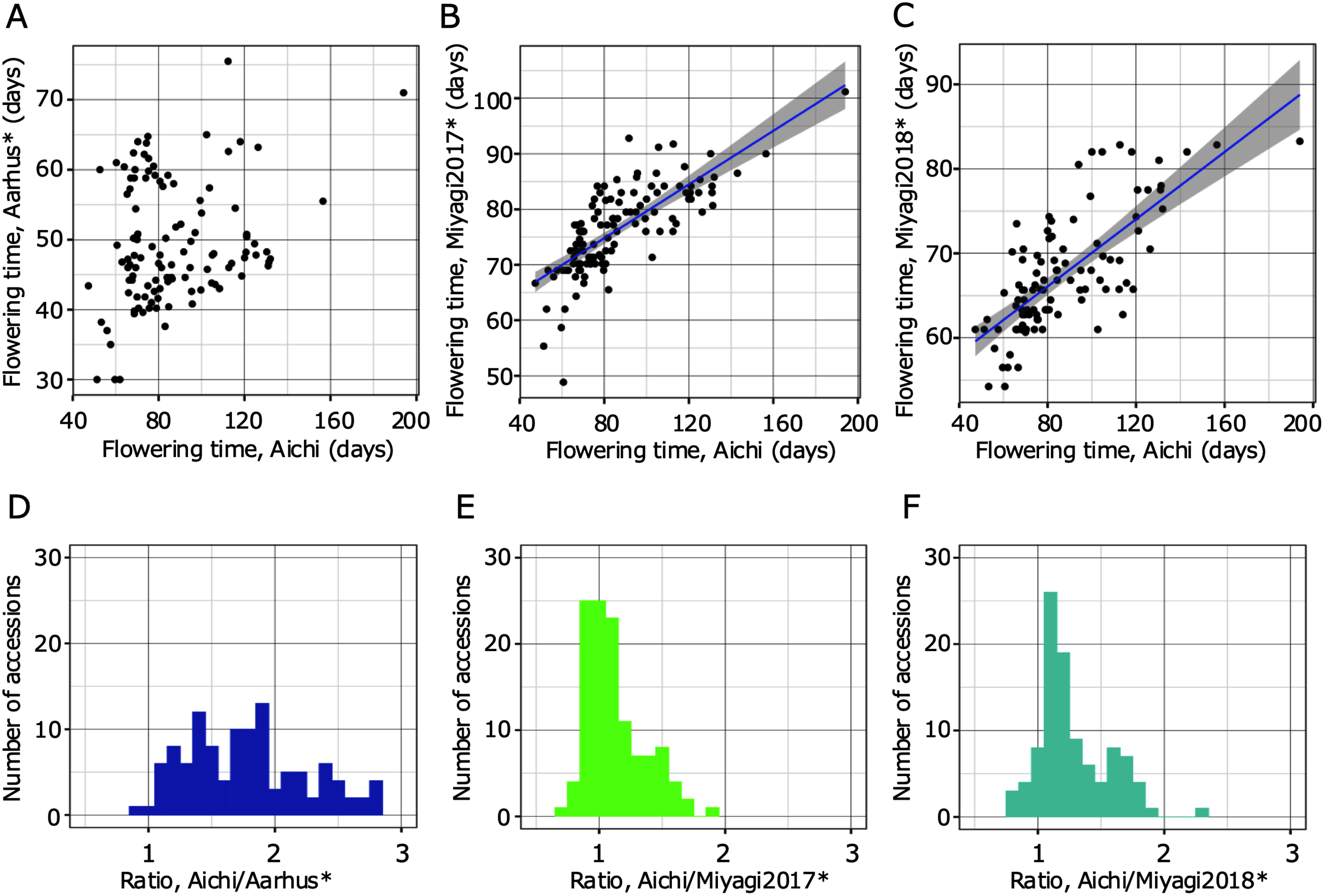
Figure 2. Correlations and distribution of ratios between flowering time at Aichi and Aarhus and Miyagi in 2017 and 2018. A–C show the plots for two flowering time averages. Each flowering time is shown on the vertical and horizontal axes (days). The blue line and gray area represent the regression line and the 95% confidence interval, respectively. D–F represent distributions of flowering time ratios. The ratio is shown on horizontal axis, and number of accessions is shown on vertical axis. *The flowering time data at Aarhus and Miyagi were obtained in Shah et al. (2020).

**Figure 3:**
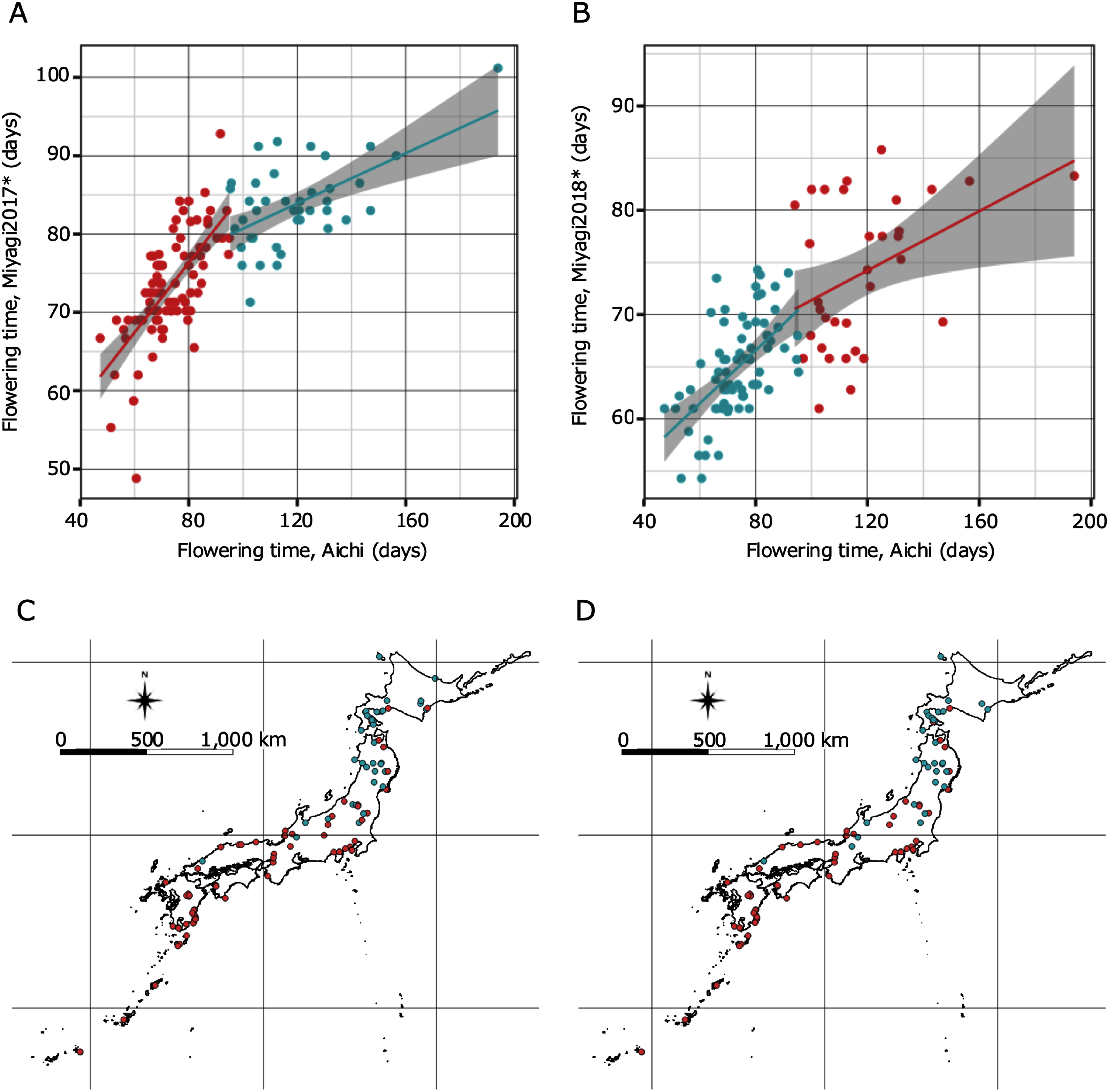
Figure 3. Result of clustering analysis for pairs of flowering time data and geographical distribution. A and B show the plots for two flowering time averages with colors based on the k-means clustering. Each flowering time is shown on the vertical and horizontal axes (days). The pink and blue lines and gray area represent the regression line and the 95% confidence interval, respectively. C and D represent geographical distributions of accessions with colors based on the k-means clustering. *The flowering time data at Miyagi were obtained in Shah et al. (2020).

The date when the first flower was fully opened was recorded, and the mean of three individuals of the same accession was used as the date for that accession. The number of days from germination to the first flower opening was documented, and correlations were determined between the latitude of collection sites of wild accessions and average flowering days in each accession, and between flowering time in Aichi and a greenhouse in Aarhus, and a field in Miyagi in 2017 and 2018, using the R version 3.2.0 (R Core Team 2015). The flowering time data at Aarhus and Miyagi were obtained in Shah et al. (2020). Regarding the pairs of Aichi and each of Miyagi in 2017 and 2018 obtained in Shah et al. (2020), cluster analysis was conducted with k-means method using R version 3.2.0 (R Core Team 2015). Using the R package ggplot2 (Wickham 2016), plots and histograms were drawn (Figures 1, 2, 3). Figure 3C, D show the locality information of accessions with collection site information drawn with colors based on the clustering results using QGIS 2.18.2 (QGIS Development Team, 2016).

### Genome-wide association mapping for flowering time

We conducted genome-wide association analyses on the genotype data (Shah et al. 2020) using an accelerated mixed model (AMM; Kang et al. 2010; Seren et al. 2012) with PyGWAS v1.7.4 (https://github.com/timeu/PyGWAS). The *L. japonicus* genome assembly build 3.0 (Sato et al. 2008) was used as the genome reference, and this facilitates comparison with the results of previous study, Shah et al. (2020). The non-trans formed phenotype variables were used. Flowering time phenotypes tend to be correlated with genetic structures, and analysis by GWA is likely to detect false positives; therefore, we adopted AMM, which is a model that considers population clusters and structures. To set the threshold, we adopted permutation test with 1,000 randomized data. Each *p*-value were set to have less than 5 or 10% false-positives. Manhattan and Q–Q (quantile–quantile) plots were drawn for all analyses using the R package qqman (Turner 2018).

Flowering time data were gathered from a greenhouse at Aichi under the same conditions, individuals with no flowers were removed, and 121 out of 132 wild accessions were chosen for GWA analyses (sampling points and flowering days of used accessions are shown in Supplementary Table S1). In order to consider how to handle non-flowered accessions, GWA analysis for a tentative data set in which non-flowering individuals were assigned 250 flowering days was also conducted. As the result, in addition to the regions detected in the analysis excluding the non-flowering accessions, the associations in many other SNPs were highly evaluated. Therefore, we consider that the regions detected in this result for the tentative data contained false positives, and adopted the flowering time result without the non-flowered individuals for GWA analysis. Genes around the SNPs beyond the significance threshold were selected as possible candidates.

In addition to a single flowering time at Aichi, GWA analyses were conducted for the ratio of the two average flowering times to detect genetic factors for differences in critical day length shown in the comparison of flowering time data. The ratio was calculated with the average flowering time of common 107, and 101 accessions at Aichi, and each of the flowering data at Miyagi in 2017 and 2018 as previously described by Shah et al. (2020) (flowering day ratios of accessions used are shown in Supplementary Table S1). Genes around the SNPs beyond the significance threshold were selected as possible candidates because SNPs did not show lower *p*-values on the whole of these analyses.

### Annotation of candidate genes

The position of SNPs detected by GWA analysis allowed for amino acid sequences of candidate genes to be determined from the *L. japonicus* genome assembly build 3.0, using bedtools2 v2.26.0 (Quinlan and Hall 2010). Genes located 10 kg base pairs (kbp) upstream or downstream (20 kbp in total) of SNPs were detected and considered in linkage disequilibrium (Shah et al. 2020). To further annotate candidate genes, the BLASTP function of BLAST 2.2.26 was employed (Altschul et al. 1990) on the website (https://blast.ncbi.nlm.nih.gov/Blast.cgi (Accessed Dec 27, 2023)). The top hit genes from BLAST with the amino acid sequences coded by known flowering time genes were used as a priori for *L. japonicus*.

### Flowering time prediction with candidate SNPs

We compared flowering time at Aichi and genotype combinations in 121 accessions to evaluate the degree of flowering time variation contributed by genotypes of the detected SNPs. To impute missing data in the SNP set, BEAGLE 5.1, (Browning et al. 2018) was used with default settings. To select candidate SNPs, those on chromosome 0 were first removed because this group contains short genome sequence fragments (<10 kbp) that were not assembled in chromosomes 1 to 6. Next, the SNP with the lowest *p*-value in the GWA analysis of each linkage SNP set was selected based on linkage disequilibrium (LD) decay estimation (<0.5). The top ten non-linkage SNPs were used to predict flowering time. Genotypes of the top SNPs with low *p*-values in the GWA analysis were added as possible candidates in the linear regression models; each coefficient of determination value (*R*^2^) for multiple regression analyses was calculated using R version 3.2.0. The formulae were also estimated in the linear regression analysis, and the predicted flowering time was calculated using the formulae. The norms of flowering time for each combination of genotypes were shown using 1 and 2 SNPs. The genotype distributions of the top SNPs with collection site information were drawn by QGIS 2.18.2, with coastline data received from the Japan Meteorological Agency.

To avoid the possibility that the results reflected the genetic structure, permutation tests were conducted for the 1st SNP and top two SNP combination. For the permutation of the 1st SNP genotype and flowering time correlation, 1,000 SNPs (minor allele frequency (MAC)=16, same as the 1st SNP) were randomly selected from chromosome 1 to 6. Regarding the permutation of top 2 SNPs combination, 1,000 combinations were made from SNPs (MAC=16 and 13, same as the 1st and 2nd SNPs) randomly selected from chromosome 1 to 6, respectively. For all SNPs and SNP combinations, coefficients of determination value (*R*^2^) for multiple regression analyses were calculated and histograms were drawn using R version 3.2.0, respectively.

## Results

### Flowering time variation in *L. japonicus* in Japan

The flowering times of 132 Japanese wild accessions are summarized in Supplementary Table S1, along with their original data. According to Shah et al. (2020), the first flowering was observed in MG-105 (Miyagi; 38°27′36″ N, 141°05′24″ E) on March 12, 2014, 47 days after germination. In eight accessions (MG-9, 32, 35, 94, 95, 98, 99, and 125), no flowering occurred within 200 days (to August 22, 2014). However, these plants continued growing and the shoots were a sufficient size for flowering; some larger than those of the early flowering accessions. No further studies were conducted on these plants, and they were categorized as “not flowered” in Supplementary Table S1. The mean flowering time of the three individual plants in each accession was calculated, and further analyses were conducted.

Based on the 120 accessions with flowering time data, a broad-sense heritability of 0.89 was calculated using ANOVA. In 115 of the 120 wild accessions (the locality information for two accessions was lost), a strong correlation was observed between latitude and flowering time, as shown in Figure 1 (*R*=0.67, *p*<0.01), with accessions from southern Japan tending to flower earlier. Compared to the flowering time variation reported in the Denmark greenhouse and Miyagi field (Shah et al. 2020), our data showed a more remarkable difference in flowering time among accessions, and a clear correlation between flowering time and latitude (Figure 1). Flowering time variations under four different conditions revealed larger differences with a shorter day length (Figure 1). The Danish greenhouse under constant long-day conditions showed smaller differences in flowering time among accessions and a weaker correlation with latitude than the others tested (*R*=0.076, *p*<0.05). The correlation coefficient values for the field at Miyagi in 2017 and 2018 between flowering time variations, and latitude were 0.57, 0.53, respectively (*p*<0.05). Correlation tests between flowering time variations under different conditions in Aichi and Miyagi in both 2017 and 2018 were relatively strongly correlated (*R*=0.75, 0.73, respectively, *p*<0.05; Figure 2B, C). Figure 2E, F show that most accessions had ratios near 1.0, with some greater than of 1.0. Many accessions showed the same flowering time between pairs of conditions and flowering of those delayed in the greenhouse at Aichi compared with the flowering time in the field at Miyagi. Clustering analyses showed that accessions could be divided into two groups with the above-mentioned tendencies (Figure 3A, B). The accessions grouped in cluster2 were mainly originated in Northern area containing Tohoku and Hokkaido areas (Figure 3C, D).

### GWA analyses for flowering time

**Figure 4:**
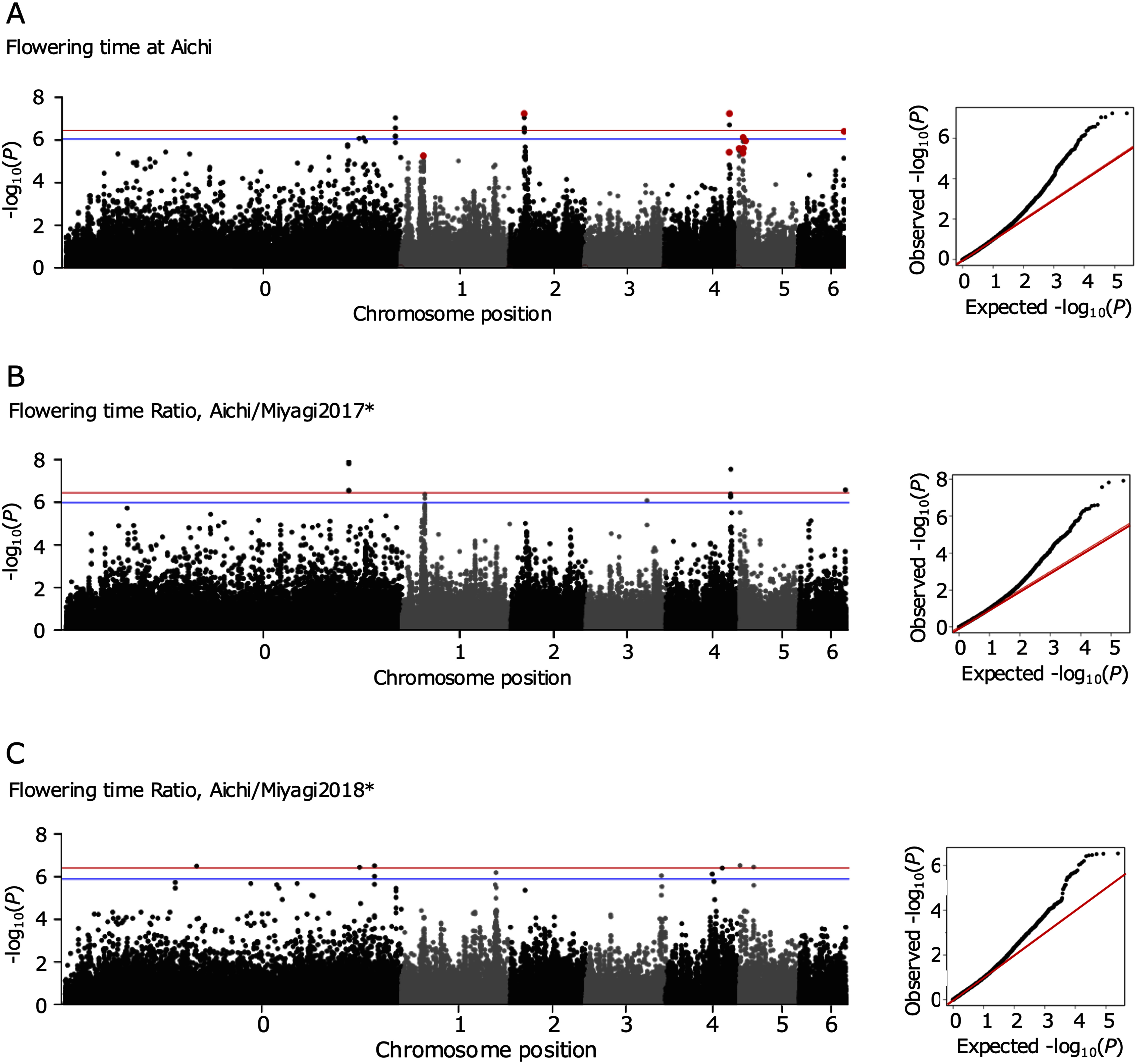
Figure 4. Manhattan plots of genome-wide association analyses for flowering time variation and flowering time ratio. Manhattan plots are shown on the left side. The vertical axis and the horizontal axis show the −log_10_(*p*-values) and chromosome positions, respectively. The positions and −log_10_(*p*-values) for each single nucleotide polymorphism (SNP) were plotted. The (quantile–quantile) Q–Q plots are shown on the right side. The red and blue lines in Manhattan plots indicate the threshold set by permutation tests with 5 and 10% false-positives. (A) The plots show the results of GWAs for flowering time in Aichi. (B, C) Each plot shows the flowering time ratio between Aichi and each of Miyagi 2017, and 2018. *The flowering time data at Miyagi were obtained in Shah et al. (2020).

GWA analyses for flowering time variation in the Aichi greenhouse showed several peaks in Manhattan plots. According to the permutation test, the thresholds were set at −log(*p*-value)=6.46, 6.05 for 5 and 10% false-positives, respectively (Supplementary Figure S1A). Figure 4A shows the result of 124 921 SNPs (MAF≥0.1), which included 4 and 10 peaks beyond the threshold of 5 and 10% false-positives, respectively. These peaks were located on chromosomes 0, 2, 4, 5 and 6, respectively. Based on linkage disequilibrium of 10 kbp, 36 genes were found around the detected 12 SNPs; 14 were NULL or repeat genes that serve no function, and 22 protein-coding genes were identified by BLAST (Table 1).

**Table 1:** Table 1. The result of gene annotation for the candidate genes on the basis of the GWA analysis for flowering time in Aichi.

Linkage group	Detected SNP^a^	−log_10_ (*p*-value)^b^	Gene ID *L. japonicus*	Description^c^	Protein ID *A. thaliana*/*G. max*	Query cover (%)	Identity (%)	Reference
Chr0	168288660^†^	6.07	Lj0g3v0322259	No hits	—	—	—	
				factor Xa inhibitor BuXI	CAA0332735.1	88	38.67	
	170744966^†^	6.10	Lj0g3v0326649	RING/U-box superfamily protein	NP_001330419.1	100	38.95	
				probable E3 ubiquitin-protein ligase RHG1A isoform X2	XP_006578031.1	100	76.89	
	189080529^†^	6.19	Lj0g3v0359189	basic helix-loop-helix (bHLH) DNA-binding superfamily protein	NP_200279.1	100	72.92	
				Transcription factor ILR3	KAH1247754.1	100	90.43	
Chr2	**8439786**	7.24	Lj2g3v0621090	unnamed protein product	CAD5315980.1	93	30.75	
				hypothetical protein GYH30_025268	KAH1043336.1	99	66.23	
	8482295	7.05	Lj2g3v0621130	No hits	—	—	—	
				No hits	—	—	—	
			Lj2g3v0621140	No hits	—	—	—	
				No hits	—	—	—	
	8548374^†^	6.37	Lj2g3v0621310	eukaryotic release factor 1-3	NP_189295.3	99	90.30	
				eukaryotic peptide chain release factor subunit 1-3	XP_006587430.1	100	96.34	
			Lj2g3v0621320	forever young oxidoreductase	AAG44120.1	99	69.62	
				dehydrogenase/reductase SDR family member FEY	XP_003534088.1	98	83.71	
	8569194	6.57	Lj2g3v0621330	No hits	—	—	—	
				No hits	—	—	—	
	8569194, 8580319	6.57	Lj2g3v0621350	unnamed protein product	CAA0278661.1	99	33.56	
				kinase-interacting family protein-like isoform 1	NP_001401645.1	100	78.17	
			Lj2g3v0621360	No hits	—	—	—	
				No hits	—	—	—	
Chr4	37258674, **37258719**	7.24	Lj4g3v2785700	small nuclear ribonucleoprotein, putative	AAM63846.1	100	81.06	Swaraz et al. 2011
				Small nuclear ribonucleoprotein SmD3b-like	NP_001237722.1	100	93.94	
			Lj4g3v2785710	Nucleotide-sugar transporter family protein	NP_001328903.1	99	78.49	
				UDP-rhamnose/UDP-galactose transporter 4	XP_006585315.1	99	92.44	
			Lj4g3v2785730	mediator of RNA polymerase II transcription subunit-like protein	NP_190854.1	87	68.47	
				mediator of RNA polymerase II transcription subunit 28	XP_003525092.1	100	88.89	
			Lj4g3v2785740	alkaline/neutral invertase	NP_001332722.1	74	87.42	Wang et al. 2022
				Alkaline/neutral invertase E, chloroplastic	KAH1237155.1	100	86.69	
Chr5	2762060^†^	6.12	Lj5g3v0296260	WPP1	OAO90978.1	78	57.89	
				MFP1 attachment factor 1	XP_003544478.1	89	59.84	
			Lj5g3v0296280	No hits	—	—	—	
				CLE11 protein	ADW77265.1	100	63.22	
Chr6	**26476140** ^†^	6.41	Lj6g3v2274460	ARM repeat superfamily protein	NP_194494.2	100	78.93	
				importin-4 isoform X2	XP_003547537.1	100	92.85	Kevei et al. 2007
			Lj6g3v2274470	senescence associated gene 18	NP_177275.1	100	70.03	
				uncharacterized protein LOC100816173	XP_003547538.1	100	84.72	
			Lj6g3v2274490	potassium transport 2/3	OAO98451.1	99	66.79	Held et al. 2011
				potassium channel AKT2/3 isoform X1	XP_003532990.1	99	84.30	
			Lj6g3v2274500	unnamed protein product	CAA0340358.1	96	48.85	
				uncharacterized protein LOC100818401	XP_003539403.2	100	85.99	
			Lj6g3v2274510	unnamed protein product	VYS46852.1	96	46.71	
				uncharacterized protein LOC100811367	XP_006586379.2	100	78.62	

The *E*-values for all of these results were under 1.0e-4. ^a^ SNPs in bold were included in seleced 10 SNPs for calculation of coefficient of determination. ^b^ If there are multiple, indicate the highest value. ^c^ The upper and bottom descriptions show results for *Arabidopsis thaliana* and Glycine max. ^†^ SNPs below 5% FDR threshold and beyond 10% FDR threshold.

GWA analyses for the flowering time ratio presented peaks in Manhattan plots beyond the thresholds. Figure 4B, C relate the results for flowering time ratio between Aichi and Miyagi in 2017 and 2018 with 126 576, 124 022 SNPs (MAF≥0.1), respectively. The ratio of two flowering time data are further presented as “Aichi/Miyagi2017”, and “Aichi/Miyagi2018”. By the permutation test for Aichi/Miyagi2017 and Aichi/Miyagi2018, the thresholds were set at −log(*p*-value)=6.47, 6.0, and 6.43, 5.91 for 5 and 10% false-positives, respectively (Supplementary Figure S1B, C). In the analyses for flowering time ratio of Aichi/Miyagi2017 and 2018, 6 and 9 peaks were detected beyond the threshold of 10% false-positives, respectively, with each 23 protein-coding genes located around them (Figure 4B, C and Supplementary Table S2). Two regions on chromosome 4 and 6, and one region on chromosome 0 detected in the analysis for Aichi/Miyagi2017 and 2018, respectively, overlapped with those from Aichi (Supplementary Table S2). Nine and two of the candidate genes were present in these regions (Supplementary Table S2). The remaining candidate genes were uniquely detected in each analysis.

### Gene annotation of candidate genes

Gene annotation with peptide sequences was conducted for protein-coding candidate genes associated with flowering time at Aichi, flowering time ratio, and two environmental factors. Table 1 shows the results for candidate genes associated with flowering time variations in Aichi. Although these candidates had several orthologues of unknown genes or those with no hit results, there were also two orthologues of known flowering time genes (Table 1), which were coded for the protein sequences small nuclear ribonucleoprotein-like (SmD3b-like; Swaraz et al. 2011) and potassium channel AKT2/3 (AKT2/3; Held et al. 2011). The known flowering time genes showed altered flowering time phenotypes in their mutant lines in all previous studies (Held et al. 2011; Swaraz et al. 2011). In addition, although no references show a direct relation, candidate genes contained another orthologue that might be related to flowering time regulation, *importin-4* (Kevei et al. 2007). In addition to these genes, *the mediator of RNA polymerase II transcription subunit 28* (*MED28*) is a gene family that contains several flowering time-related genes, for example *MED8*, *MED12*, *MED13*, *MED15*, *MED16*, *MED17*, *MED18*, *MED20a*, and *MED25* (Buendía-Monreal and Gillmor 2016; Yao et al. 2019). These mediator subunit gene mutants exhibit a late-flowering phenotype. All of the detected protein-coding genes on chromosome 4, including the orthologues of *SmD3b-like* and *MED28*, were located in the 20 kbp region around the SNPs of 37 258 719 bp and 37 258 674 bp, which were the most strongly associated with flowering time variation. In particular, for *MED28*, both SNPs were located in the gene region, whereas *AKT2*/*3* and *importin-4* were located in a region around the SNP of 26 476 140 bp on chromosome 6.

Regarding the results for flowering time ratio, known flowering time genes were contained in the candidate genes (Supplementary Table S2). Candidate genes for Aichi/Miyagi2017 and Aichi/Miyagi2018 shared nine and one genes with the candidates for flowering time variation in Aichi (Supplementary Table S2), respectively. These genes contained known flowering time genes, *SmD3b-like*, and *AKT2*/*3*. Apart from the shared genes mentioned, there was one other orthologue of known flowering time gene in the results for Aichi/Miyagi2018, which were located on chromosome 4. The gene was orthologue of *WDR5A* (Jiang et al. 2009). It has been reported that *WDR5A* is an activator which mediates *Flowering Lotus C* (*FLC*) upregulation by *FRIGIDA* (*FRI*) and related to flowering time regulation in *A. thaliana* (Jiang et al. 2009). Besides these orthologues of flowering time related genes, several genes which belong to the protein families containing flowering time related ones were detected (Supplementary Table S2). One of them is an orthologue coding alpha/beta-Hydrolases superfamily protein on chromosome 0 and it is known that one of the family members negatively regulated salt tolerance but promotes flowering in rice (Xiang et al. 2022). Other than this candidate, an orthologue coding NAD(P)-binding Rossmann-fold superfamily protein which was located on chromosome 3. According to Xing et al. (2014), *SDR6* gene contains a NAD(P)-binding domain and *sdr6* mutants displayed later flowering time than wild type in *A. thaliana*. On the basis of expression levels of the key genes, it is suggested that *SDR6* may be involved in the autonomous flowering pathway (Xing et al. 2014).

### Modeling of flowering time with SNPs that had lower *p*-values in the genome-wide association analysis for flowering time

**Figure 5:**
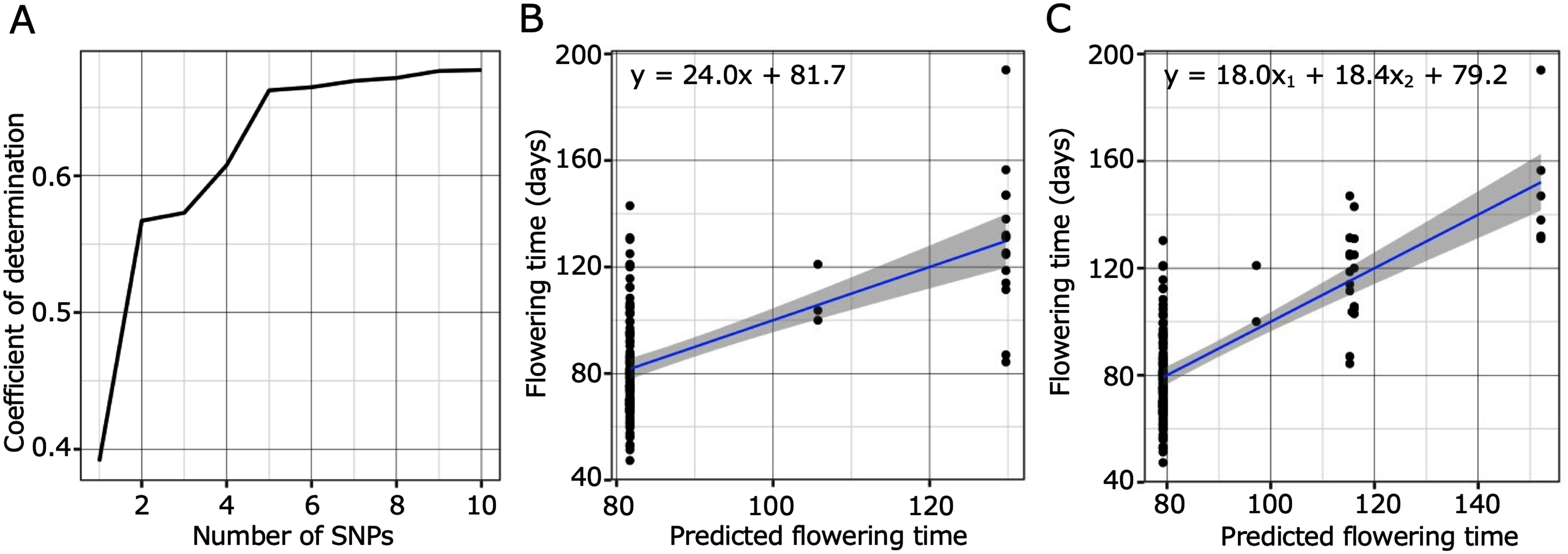
Figure 5. Correlation between flowering time variation and combination of detected SNPs by genome-wide association analysis for flowering time. (A) The vertical axis and the horizontal axis show the coefficient of determination and the number of single nucleotide polymorphisms (SNPs) contained in the SNP combinations, respectively. (B, C) The vertical axis and the horizontal axis show flowering time (days) and predicted flowering time for cases with the 1st and 2nd genotypes, respectively. Plots denote flowering time and predicted flowering time for each accession. Predicted flowering time was calculated by the estimated formula shown in each figure.

**Figure 6:**
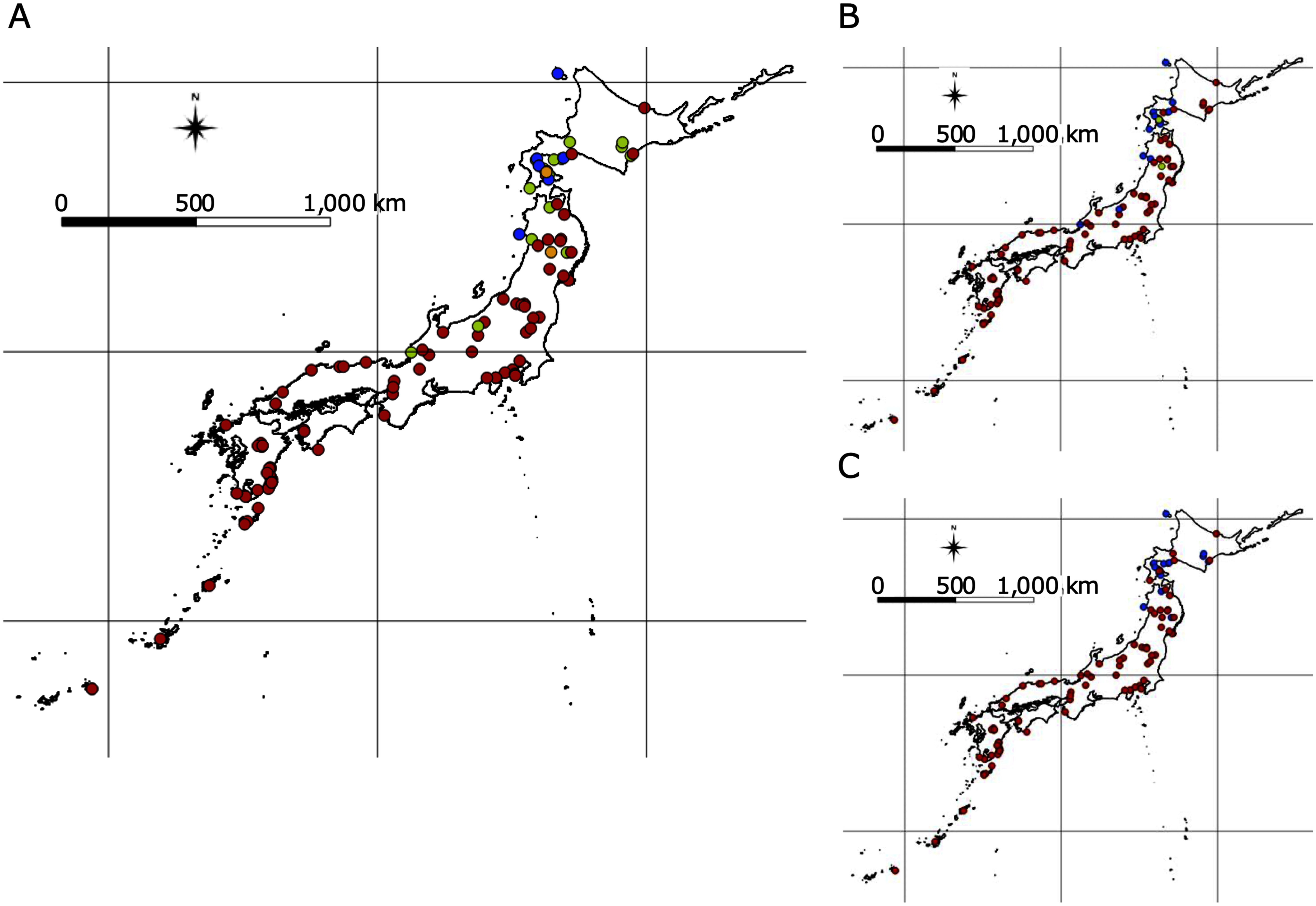
Figure 6. Genotype distribution for selected SNPs in correlation test with flowering time variation. Each color of the circles denotes the locations of accessions with genotypes of single nucleotide polymorphisms (SNPs) selected by correlation with flowering time variation. The genotype distribution in which the top two SNPs were taken into account is shown in A. Red and blue circles show early- and late-flowering genotypes, respectively. Green circles show accessions in which one of the two SNPs was fixed in the early flowering stage and the other had the late-flowering genotype. Yellow circles show accessions in which one of the two SNPs had the hetero genotype and the other had the early flowering genotype. The genotype distributions of the 1st and 2nd SNPs, Chr4 37258719 and Chr2 8439786, are shown in B and C, respectively. Red and blue circles indicate early- and late-flowering genotypes, respectively. Green circles indicate heterotypes.

Based on the result of GWA analysis for flowering time variation at Aichi, the 10 selected SNPs are listed in Supplementary Table S3. The most strongly detected SNP, 37 258 719 bp on chromosome 4 showed an *R*^2^-value was 0.391. With the addition of the top 2 SNPs, the value reached 0.567 (Figure 5A and Supplementary Table S3). In both cases, all added SNPs were significantly selected as explanatory variables for flowering time variation (*p*<0.001). According to the results of permutation tests, with top 5%, *R*^2^ values were 0.167 and 0.18 with randomly selected SNP and two SNPs combination with same allele frequencies as the detected top 2 SNPs, respectively (Supplementary Figure S2). Figure 5B, C shows the plots for flowering time and predicted flowering time for the top 1 and 2 genotypes, respectively. The predicted flowering time was calculated using the estimated formula shown in Figure 5B, C. Furthermore, the *R*^2^-value reached approximately 0.66, using the top 5 SNPs (Figure 5A), and the values leveled off at 0.66–0.67, with more than 5 SNPs, in which case the 1st, 2nd, and 5th SNPs were selected as significant explanatory variables. Genotype distributions for each of 1st and 2nd SNPs are shown in Figure 6B, C, and the accumulation of the top 2 SNPs is shown in Figure 6A, which indicates that there were geographic tendencies in each SNP distribution. For both of these SNPs, the genotypes shared by the late-flowering accessions were distributed in areas north of the Hokuriku region. In Shah et al. (2020), wild accessions originating from Japan were clustered based on genome-wide nucleotide polymorphism information, and one of the three groups, subpopulation 3, included accessions north of the Kanto and Hokuriku regions. Within this subpopulation 3, which contains genetically more closely related accessions, we examined whether there were differences in flowering time between the genotypes of the top 2 SNPs to verify the effects of these SNPs and the detected candidate genes on the phenotype. Comparing flowering time between genotypes of the 1st and 2nd SNPs in subpopulation 3 shown in Shah et al. (2020), accessions with the alternative genotype flowered significantly later than accessions with the reference genotype at the 2nd SNP (Supplementary Figure S3). In addition, there were 4 and 1 protein-coding genes around these SNPs, respectively (Table 1), one of which around the 1st SNP was *the SmD3b-like* orthologue, which is a known flowering time-related gene.

## Discussion

Flowering time measurements in this study showed substantial differences in *L. japonicus* under a shorter day length than in a previous study (Shah et al. 2020). Flowering time variation may be associated with environmental factors that vary by latitude, such as temperature and day length, and accessions may be able to adapt to the different habitats through flowering time. Previous studies have revealed that flowering time is generally controlled by external factors such as day length and temperature (Andrés and Coupland 2012; Song et al. 2013). In this study, we obtained new data on flowering time under shorter day length conditions and compared the correlations observed between flowering time variations and sampled latitudes, demonstrating that there were larger differences and stronger correlations under shorter day lengths. For example, the differences in flowering time in Aichi and Aarhus in averages between the earliest and latest flowering accessions were 146.67 and 45.5 days, respectively. Considering that *L. japonicus* is a long-day plant, the variance difference suggests that the extremely long-day conditions in Aarhus uniformly promoted flowering in this species, even in late flowering accessions under shorter day lengths; therefore, flowering time and latitude were not correlated with flowering variation in this region. In Miyagi, although correlations existed between the flowering time variations and latitude, the differences in average flowering time between the earliest and latest flowering accessions were 52.4 and 31.5 days, in 2017 and 2018, respectively; this is considerably shorter than the results from Aichi, of 146.67 days. By comparing the data newly obtained in this study under shorter day length with data under long day conditions obtained in the previous study, it was suggested that day length was one of the most important signals for *L. japonicus* flowering time control. Regarding the 2 years of data in Miyagi, obtained in Shah et al. (2020) , the observed correlations showed almost the same level, while a vertical difference of several days in the two regression lines implies that a gap in the planting season between the two experiments might affect flowering days. Despite the fact that sowing and planting were performed 3 and 2 weeks earlier, respectively, in 2018, and that this experiment started under longer day length conditions than 2017, shorter flowering days were observed in 2018. However, if there are other conceivable reasons than a gap in planting seasons, temperature can be a factor. There were 1.6 and 4.4°C differences in the average and maximum temperatures, respectively, in June between these years, according to data from the Japan Meteorological Agency. Day length is suggested to be a significant factor for degrees of flowering time differences among accessions, based on comparisons of several conditions, and differences between years in Miyagi imply that day length and/or temperature possibly affected the flowering days.

The broad-sense heritability for flowering time variation in this study reached 0.89 for all flowered individuals in 120 out of 132 accessions, as calculated by analysis of variance, and this variation was considered to be largely due to genetic factors. Large geographic distances between wild populations, the wide variations in flowering time, and the occurrence of non-flowering accessions for more than 200 days, indicate that intraspecific differentiation occurs among Japanese *L. japonicus*. There is a possibility of reproductive isolation because these factors could preclude crossing opportunities between some ecotypes of this species. The flowering time variations showed continuous values, which suggested that it was regulated by several factors. Genetic control of flowering time in plant species is complex, and genetic factors that induce flowering time variation could contain genetic heterogeneities or allelic heterogeneities. In this study, the results of GWA analyses for flowering time variation showed several peaks, which implies that there may be numerous genetic factors and/or genetic or allelic heterogeneities in the flowering time pathway for this species.

GWA analyses for flowering time in Aichi detected candidate genes containing orthologues of known flowering time genes. Compared to the GWA analysis results for flowering time variations in a previous study (Shah et al. 2020), our analysis detected different candidate genes. In the previous study, there were peaks for each flowering time variation, however, these regions were different from those detected in this study. In particular, the regions detected in this study showed stronger associations with flowering time variation at Aichi than the analyses for the flowering time under longer day length in previous study. This could relate to differences in flowering time tendencies or the accessions used for the analysis. Sixteen protein-coding genes were detected, two being orthologues of known flowering time genes (Figure 4A and Table 1), coding SmD3b-like and AKT2/3 (Held et al. 2011) which have been shown to have functions associated with flowering time phenotypes in other species, particularly *AKT2*/*3*, an orthologue in the common bean that has been associated with flowering time (Raggi et al. 2019) that could be influential in maintaining flowering time variation. In addition to these two genes, there were two others that could be related to flowering time regulation, depending on their functions. One of the genes, Lj6g3v2274460, is an orthologue of *importin-4* (Table 1); this gene is known to be involved in targeting photoreceptors (Kevei et al. 2007) and may also regulate flowering time. The other is Lj4g3v2785730, an orthologue of *MED28*, which encodes one of the mediator subunits, and certain mediator subunit mutants of *Arabidopsis thaliana* showed late flowering phenotypes (Buendía-Monreal and Gillmor 2016; Yao et al. 2019). Candidate genes, especially the above genes and orthologues of known flowering time genes, could contribute to the regulation of flowering time in this species and may even promote intraspecific differentiation and local adaptation via flowering time alterations. Among other legumes, there are no previous studies on the interactions between these genes and flowering time control. In future studies, we plan to conduct experiments with mutant or transformation lines to reveal the relationships between these genes and flowering time control in this species and possibly in legumes.

Besides flowering time under the shorter day length, in this study, we conducted GWA analyses using flowering time ratio as a new attempt. Orthologues of known flowering time genes were detected by GWA analyses for the ratio between flowering time in Aichi and Miyagi in 2017. Compared with the results for flowering time in Aichi, a few peaks were emphasized and exceeded the threshold with the ratios of flowering time data. This implies that these ratio analyses could reflect the differences in flowering time phenotypes between conditions and the candidate genes related to these differences. Common candidates, including *SmD3b-like* and *AKT2*/*3*, would be more likely candidates because these genes were detected universally in the analyses of both single flowering time data and ratios. With regard to the ratio between flowering time at Aichi and Miyagi in 2017 and 2018 (Figure 2E, F), we found that flowering time in the greenhouse at Aichi differed among the accessions that showed comparable phenotypes in the field at Miyagi (Figure 2B, C). In addition, this tendency was particularly prevalent in certain accessions derived from northern habitats. This result implies that other factors affect flowering time control in northern varieties, such as adequate vegetative growth terms for each accession. With adequate growth, it is likely that *L. japonicus* from the northern part of Japan has adapted to the environment with severe cold in winter and a short summer, which is suitable for this species to bloom. In GWA analyses for these ratios, Aichi/Miyagi2017 and Aichi/Miyagi2018, candidate genes would reflect the relationships with vegetative growth. Under the assumption that a longer vegetative growth term would be required for some of the accessions, specific candidate genes for these ratios, including orthologue of *WDR5A* may affect vegetative growth term length and flowering time control in *L. japonicus*. In *A. thaliana*, it has been reported that the *WDR5A* gene plays a role in association with H3K4 methyltransferases as a core component of COMPASS-like complex and related to control floral transition and plant development (Jiang et al. 2011). By using the ratio of flowering time data under different cultivation conditions, in this study we obtained candidate genes that may be involved in vegetative growth, including *WDR5A*. In a future study, it may be advantageous to focus on the growth term length to aid in the understanding the mechanisms of maintaining flowering time variation in this species.

In *A. thaliana*, *FRIGIDA* greatly affected the latitudinal change in flowering time (Stinchcombe et al. 2004). However, it is possible that there may be more than one gene that contributes to flowering time variation in a single species, and that they work in an additive manner. In addition, genetic factors for intraspecific flowering time variation could be species specific. The assessment of correlations between flowering time variation and combinations of the top detected SNPs in GWA analysis showed that adding the 1st to 2nd SNPs resulted in a large increase in the *R*^2^-value (Figure 5A). The results of the permutation test also show that this value is unlikely to occur by chance (Supplementary Figure S2). This result suggests that the variation in the flowering time of *L. japonicus* could be explained mainly by a small number of genetic factors. The top two SNPs, 37 258 719 bp on chromosome 4 and 8 439 786 bp on chromosome 2, were selected as significant variables for flowering time variation. The genotype distributions of each SNP of the genetic factors were along the latitude (Figure 6B, C). In addition, in subpopulation 3 presented in Shah et al. (2020), the alternative genotype of the 2nd SNP showed a significant delay in flowering time, and the alternative genotype of the 1st SNP also showed a tendency of late flowering time (Supplementary Figure S3), suggesting that the genes detected around these SNPs may contain genetic factors that contribute to the flowering time polymorphism in this species. Furthermore, the genotype of a combination of the two SNPs showed a gradual distribution along the latitude (Figure 6A). This implies that the variation in clinal flowering time in this species might be caused by a combination of multiple genes. The genes around these two SNPs included an orthologue of a known flowering time gene, *SmD3b-like* (Table 1). However, on the protein-coding genes located around these two SNPs, there were no non-synonymous changes in the SNP dataset used in this study. Since the SNP dataset was used as a marker to detect associated regions for variables, it is possible that this SNP set did not contain all of the mutations conserved among these accessions. Therefore, the observed gene expressions and our results suggest that some of these genes could be related to flowering time variation in this species. Moreover, flowering time variation may manifest due to the accumulation of small effects from many genetic factors. To understand the mechanisms of local adaptation and intraspecific differentiation in this species, it is therefore necessary in future studies to identify the genetic factors that induce these small effects.

## Description of Supplementary Files

Supplementary Table S1. Locality of accessions with collection site information and flowering days of each individual, imputed genotypes of top 10 SNPs of each accession. Flowering days for each individual and average of these at Aichi are shown in flowering time (Days) and average column, respectively. Imputed genotype column shows genotypes of each accession in top 10 SNP sites. 0 and 1, 2 mean reference homozygote and reference/alternative heterozygote, and alternative homozygote, respectively.

Supplementary Table S2. The result of gene annotation for the candidate genes on the basis of the GWA analysis for ratio of flowering time. Top hit annotation results are shown for each detected gene.

Supplementary Table S3. The coefficient of determination values for the most strongly detected 10 SNPs in GWA analysis for flowering time. The coefficient of determination values (*R*^2^) were calculated with flowering days at Aichi by adding the most strongly detected 10 SNPs in order.

Supplementary Figure S1. Histograms of permutation of GWAS analyses for flowering time and the ratio of flowering time at Okazaki Aichi and OsakiMiyagi.

Supplementary Figure S2. Histograms of permutation of model estimation.

Supplementary Figure S3. Boxplot of flowering time for each genotype of the top 2 SNPs in subpopulation 3 shown in Shah et al. (2020).

## References

[RAltschul1990] Altschul SF, Gish W, Miller W, Myers EW, Lipman DJ (1990) Basic local alignment search tool. *J Mol Biol* 215: 403–410 2231712 10.1016/S0022-2836(05)80360-2

[d67e1561] Andrés F, Coupland G (2012) The genetic basis of flowering responses to seasonal cues. *Nat Rev Genet* 13: 627–639 22898651 10.1038/nrg3291

[RAtwell2010] Atwell S, Huang YS, Vilhjálmsson BJ, Willems G, Horton M, Li Y, Meng D, Platt A, Tarone AM, Hu TT, et al. (2010) Genome-wide association study of 107 phenotypes in *Arabidopsis thaliana* inbred lines. *Nature* 465: 627–631 20336072 10.1038/nature08800 PMC3023908

[RBrowning2018] Browning BL, Zhou Y, Browning SR (2018) A one-penny imputed genome from next-generation reference panels. *Am J Hum Genet* 103: 338–348 30100085 10.1016/j.ajhg.2018.07.015 PMC6128308

[d67e1619] Buendía-Monreal M, Gillmor CS (2016) Mediator: A key regulator of plant development. *Dev Biol* 419: 7–18 27287881 10.1016/j.ydbio.2016.06.009

[RBurgarella2016] Burgarella C, Chantret N, Gay L, Prosperi J-M, Bonhomme M, Tiffin P, Young ND, Ronfort J (2016) Adaptation to climate through flowering phenology: A case study in *Medicago truncatula.* *Mol Ecol* 25: 3397–3415 27144929 10.1111/mec.13683

[RDittmar2014] Dittmar EL, Oakley CG, Ågren J, Schemske DW (2014) Flowering time QTL in natural populations of *Arabidopsis thaliana* and implications for their adaptive value. *Mol Ecol* 23: 4291–4303 25039363 10.1111/mec.12857

[RFournier2011] Fournier-Level A, Korte A, Cooper MD, Nordborg M, Schmitt J, Wilczek AM (2011) A map of local adaptation in Arabidopsis thaliana. *Science* 334: 86–89 21980109 10.1126/science.1209271

[RHall2006] Hall MC, Willis JH (2006) Divergent selection on flowering time contributes to local adaptation in *Mimulus guttatus* populations. *Evolution* 60: 2466–2477 17263109

[RHashiguchi2012] Hashiguchi M, Abe J, Aoki T, Anai T, Suzuki A, Akashi R (2012) The National BioResource Project (NBRP) *Lotus* and *Glycine* in Japan. *Breed Sci* 61: 453–461 23136485 10.1270/jsbbs.61.453 PMC3406794

[RHeld2011] Held K, Pascaud F, Eckert C, Gajdanowicz P, Hashimoto K, Corratgé-Faillie C, Offenborn JN, Lacombe B, Dreyer I, Thibaud JB, et al. (2011) Calcium-dependent modulation and plasma membrane targeting of the AKT2 potassium channel by the CBL4/CIPK6 calcium sensor/protein kinase complex. *Cell Res* 21: 1116–1130 21445098 10.1038/cr.2011.50 PMC3193494

[RHenderson2004] Henderson IR, Dean C (2004) Control of *Arabidopsis* flowering: The chill before the bloom. *Development* 131: 3829–3838 15289433 10.1242/dev.01294

[RJiang2009] Jiang D, Gu X, He Y (2009) Establishment of the winter-annual growth habit via *FRIGDA*-mediated histone methylation at *FLOWERING LOCUS C* in *Arabidopsis.* *Plant Cell* 21: 1733–1746 19567704 10.1105/tpc.109.067967 PMC2714927

[RJiang2011] Jiang D, Kong NC, Gu X, Li Z, He Y (2011) *Arabidopsis* COMPASS-like complexes mediate histone H3 lysine-4 trimethylation to control floral transition and plant development. *PLoS Genet* 7: e1001330 21423667 10.1371/journal.pgen.1001330 PMC3053346

[RKai2010] Kai S, Tanaka H, Hashiguchi M, Iwata H, Akashi R (2010) Analysis of genetic diversity and morphological traits of Japanese *Lotus japonicus* for establishment of a core collection. *Breed Sci* 60: 436–446

[RKang2010] Kang HM, Sul JH, Service SK, Zaitlen NA, Kong SY, Freimer NB, Sabatti C, Eskin E (2010) Variance component model to account for sample structure in genome-wide association studies. *Nat Genet* 42: 348–354 20208533 10.1038/ng.548 PMC3092069

[RKawaguchi2000] Kawaguchi M (2000) *Lotus japonicus* ‘Miyakojima’ MG-20: An early-flowering accession suitable for indoor handling. *J Plant Res* 113: 507–509

[RKeller2012] Keller SR, Levsen N, Olson MS, Tiffin P (2012) Local adaptation in the flowering-time gene network of balsam poplar, *populus balsamifera* L. *Mol Biol Evol* 29: 3143–3152 22513286 10.1093/molbev/mss121

[RKevei2007] Kevei E, Schafer E, Nagy F (2007) Light-regulated nucleo-cytoplasmic partitioning of phytochromes. *J Exp Bot* 58: 3113–3124 17905733 10.1093/jxb/erm145

[RLeinonen2013] Leinonen PH, Remington DL, Leppälä J, Savolainen O (2013) Genetic basis of local adaptation and flowering time variation in *Arabidopsis lyrata.* *Mol Ecol* 22: 709–723 22724431 10.1111/j.1365-294X.2012.05678.x

[RMustamin2023] Mustamin Y, Akyol TY, Gordon M, Manggabarani AM, Isomura Y, Kawamura Y, Bamba M, Williams C, Andersen SU, Sato S (2023) *FER* and *LecRK* show haplotype-dependent cold-responsiveness and mediate freezing tolerance in *Lotus japonicus.* *Plant Physiol* 191: 1138–1152 36448631 10.1093/plphys/kiac533 PMC9922393

[RQGIS2016] QGIS Development Team (2016) QGIS Geographic Information System. Open Source Geospatial Foundation Project. URL: http://www.qgis.org

[RQuinlan2010] Quinlan AR, Hall IM (2010) BEDTools: A flexible suite of utilities for comparing genomic features. *Bioinformatics* 26: 841–842 20110278 10.1093/bioinformatics/btq033 PMC2832824

[RR2015] R Core Team (2015) R: A language and environment for statistical computing. R Foundation for Statistical Computing, Vienna, Austria. URL: https://www.R-project.org/

[RRaggi2019] Raggi L, Caproni L, Carboni A, Negri V (2019) Genome-wide association study reveals candidate genes for flowering time variation in common bean (*Phaseolus vulgaris* L.). *Front Plant Sci* 10: 962 31428109 10.3389/fpls.2019.00962 PMC6689981

[RSasaki2015] Sasaki E, Zhang P, Atwell S, Meng D, Nordborg M (2015) “Missing” G x E variation controls flowering time in *Arabidopsis thaliana.* *PLoS Genet* 11: e1005597 10.1371/journal.pgen.1005597 PMC4608753 26473359

[RSato2008] Sato S, Nakamura Y, Kaneko T, Asamizu E, Kato T, Nakao M, Sasamoto S, Watanabe A, Ono A, Kawashima K, et al. (2008) Genome structure of the legume, *Lotus japonicus.* *DNA Res* 15: 227–239 18511435 10.1093/dnares/dsn008 PMC2575887

[RSchemske1978] Schemske DW, Willson MF, Melampy MN, Miller LJ, Verner L, Schemske KM, Best LB (1978) Flowering ecology of some spring woodland herbs. *Ecology* 59: 351–366

[RSeren2012] Seren U, Vilhjalmsson BJ, Horton MW, Meng D, Forai P, Huang YS, Long Q, Segura V, Nordborg M (2012) GWAPP: A web application for genome-wide association mapping in Arabidopsis. *Plant Cell* 24: 4793–4805 23277364 10.1105/tpc.112.108068 PMC3556958

[RShah2020] Shah N, Wakabayashi T, Kawamura Y, Skovbjerg CK, Wang M-Z, Mustamin Y, Isomura Y, Gupta V, Jin H, Mun T, et al. (2020) Extreme genetic signatures of local adaptation during *Lotus japonicus* colonization of Japan. *Nat Commun* 11: 253 31937774 10.1038/s41467-019-14213-y PMC6959357

[RSong2013] Song YH, Ito S, Imaizumi T (2013) Flowering time regulation: Photoperiod- and temperature-sensing in leaves. *Trends Plant Sci* 18: 575–583 23790253 10.1016/j.tplants.2013.05.003 PMC3796012

[RSrikanth2011] Srikanth A, Schmid M (2011) Regulation of flowering time: All roads lead to Rome. *Cell Mol Life Sci* 68: 2013–2037 21611891 10.1007/s00018-011-0673-y PMC11115107

[RStinchcombe2004] Stinchcombe JR, Weinig C, Ungerer M, Olsen KM, Mays C, Halldorsdottir SS, Purugganan MD, Schmitt J (2004) A latitudinal cline in flowering time in *Arabidopsis thaliana* modulated by the flowering time gene *FRIGIDA.* *Proc Natl Acad Sci USA* 101: 4712–4717 15070783 10.1073/pnas.0306401101 PMC384812

[RSwaraz2011] Swaraz AM, Park YD, Hur Y (2011) Knock-out mutations of *Arabidopsis SmD3-b* induce pleotropic phenotypes through altered transcript splicing. *Plant Sci* 180: 661–671 21421416 10.1016/j.plantsci.2011.01.011

[RTsubokura2014] Tsubokura Y, Watanabe S, Xia Z, Kanamori H, Yamagata H, Kaga A, Katayose Y, Abe J, Ishimoto M, Harada K (2014) Natural variation in the genes responsible for maturity loci *E1, E2, E3* and *E4* in soybean. *Ann Bot (Lond)* 113: 429–441 10.1093/aob/mct269 PMC3906962 24284817

[RTurner2018] Turner SD (2018) qqman: An R package for visualizing GWAS results using Q-Q and manhattan plots. *J Open Source Softw* 3: 731

[RWang2022] Wang YJ, Zhen XH, Zhou YJ, Wang YL, Hou JY, Wang X, Li RM, Liu J, Hu XW, Geng MT, et al. (2022) *MeNINV1*: An alkaline/neutral invertase gene of *Manihot esculenta*, enhanced sucrose catabolism and promoted plant vegetative growth in transgenic *Arabidopsis*. *Plants* 11: 946 35406926 10.3390/plants11070946 PMC9003190

[RWickham2016] Wickham H (2016) *ggplot2: Elegant Graphics for Data Analysis*. Springer-Verlag, New York

[RXiang2022] Xiang YH, Yu JJ, Liao B, Shan JX, Ye WW, Dong NQ, Guo T, Kan Y, Zhang H, Yang YB, et al. (2022) An α/β hydrolase family member negatively regulates salt tolerance but promotes flowering through three distinct functions in rice. *Mol Plant* 15: 1908–1930 36303433 10.1016/j.molp.2022.10.017

[RXing2014] Xing J, Zhang J, Yang P, Jiang C, Fan J, Han J, Dong J (2014) *SDR6* is involved in regulation of flowering time in *Arabidopsis thaliana.* *Plant Biotechnol (Tokyo)* 31: 133–139

[RYao2019] Yao T, Park BS, Mao HZ, Seo JS, Ohama N, Li Y, Yu N, Mustafa NFB, Huang CH, Chua NH (2019) Regulation of flowering time by SPL10/MED25 module in Arabidopsis. *New Phytol* 224: 493–504 31125430 10.1111/nph.15954

[RYu2016] Yu L-X, Liu X, Boge W, Liu X-P (2016) Genome-wide association study identifies loci for salt tolerance during germination in autotetraploid alfalfa (*Medicago sativa* L.) using genotyping-by-sequencing. *Front Plant Sci* 7: 956 27446182 10.3389/fpls.2016.00956 PMC4923157

[RZhang2015] Zhang J, Song Q, Cregan PB, Nelson RL, Wang X, Wu J, Jiang GL (2015) Genome-wide association study for flowering time, maturity dates and plant height in early maturing soybean (*Glycine max*) germplasm. *BMC Genomics* 16: 217 25887991 10.1186/s12864-015-1441-4 PMC4449526

[RZhao2011] Zhao K, Tung C-W, Eizenga GC, Wright MH, Ali ML, Price AH, Norton GJ, Islam MR, Reynolds A, Mezey J, et al. (2011) Genome-wide association mapping reveals a rich genetic architecture of complex traits in *Oryza sativa.* *Nat Commun* 2: 467 21915109 10.1038/ncomms1467 PMC3195253

