# Supplementary material for: Genome-wide association study identifies candidate genes contributing to flowering time variation in Lotus japonicus in Japan
Source: Plant Biotechnol (Tokyo). 2025 Mar 25;42(1):17–29. doi: 10.5511/plantbiotechnology.24.1023a (PMC12622898; doi:10.5511/plantbiotechnology.24.1023a)
Supplement: Supplementary Data [file plantbiotechnology-42-1-24.1023a-s001.pdf]

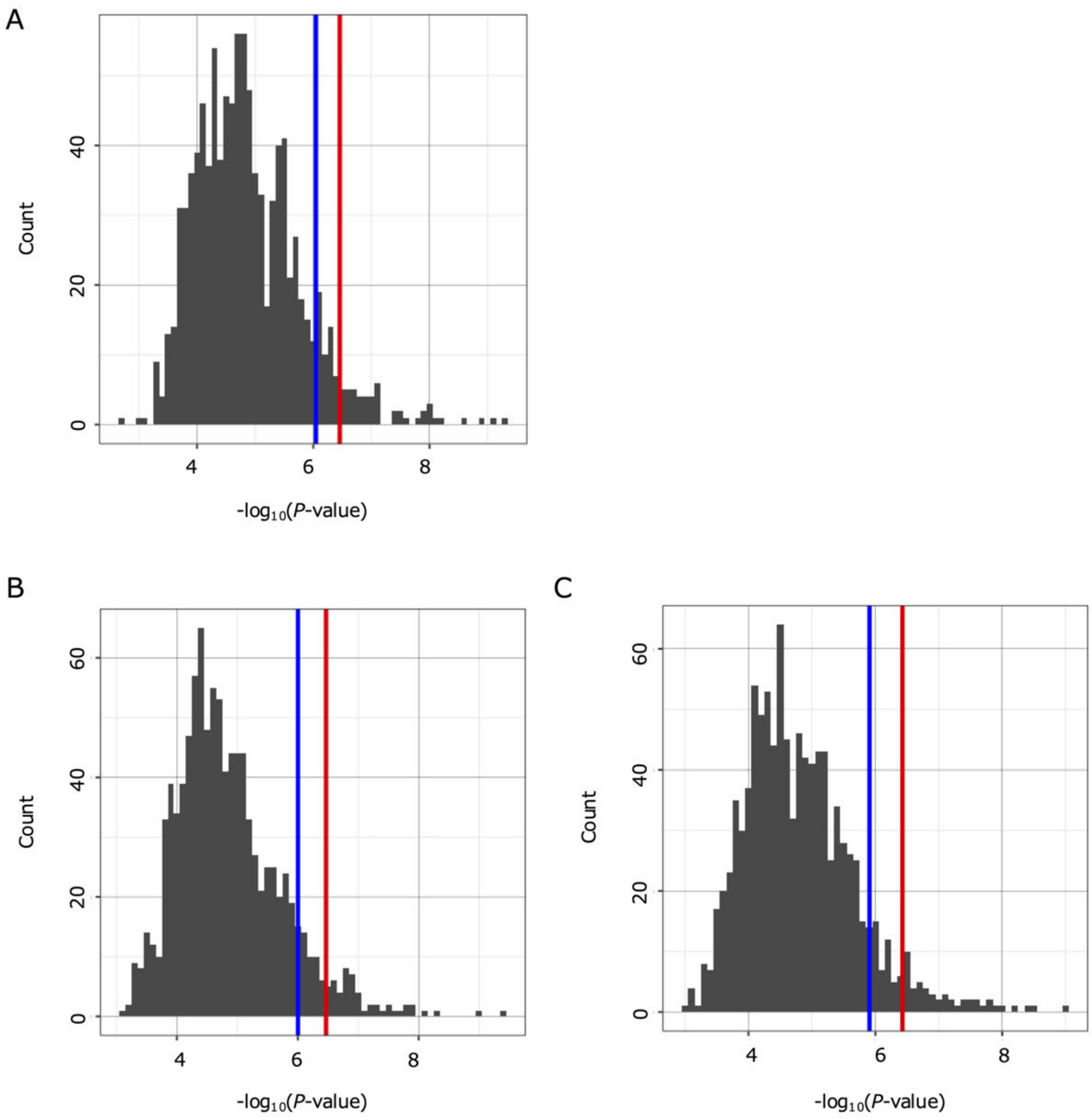

Supplementary Figure S1. Histograms of permutation of GWAS analyses for flowering time and the ratio of flowering time at Aichi and Miyagi

A–C show the histograms of the highest -$\log_{10}$(*P*-value) obtained by GWA analyses for randomized phenotype values of flowering time at Aichi and the ratio of flowering time at Aichi and Miyagi in 2017, 2018 obtained in Shah et al. 2020, respectively. The red and blue lines indicate the values shown by top 5 and 10% of the analysis results, respectively.

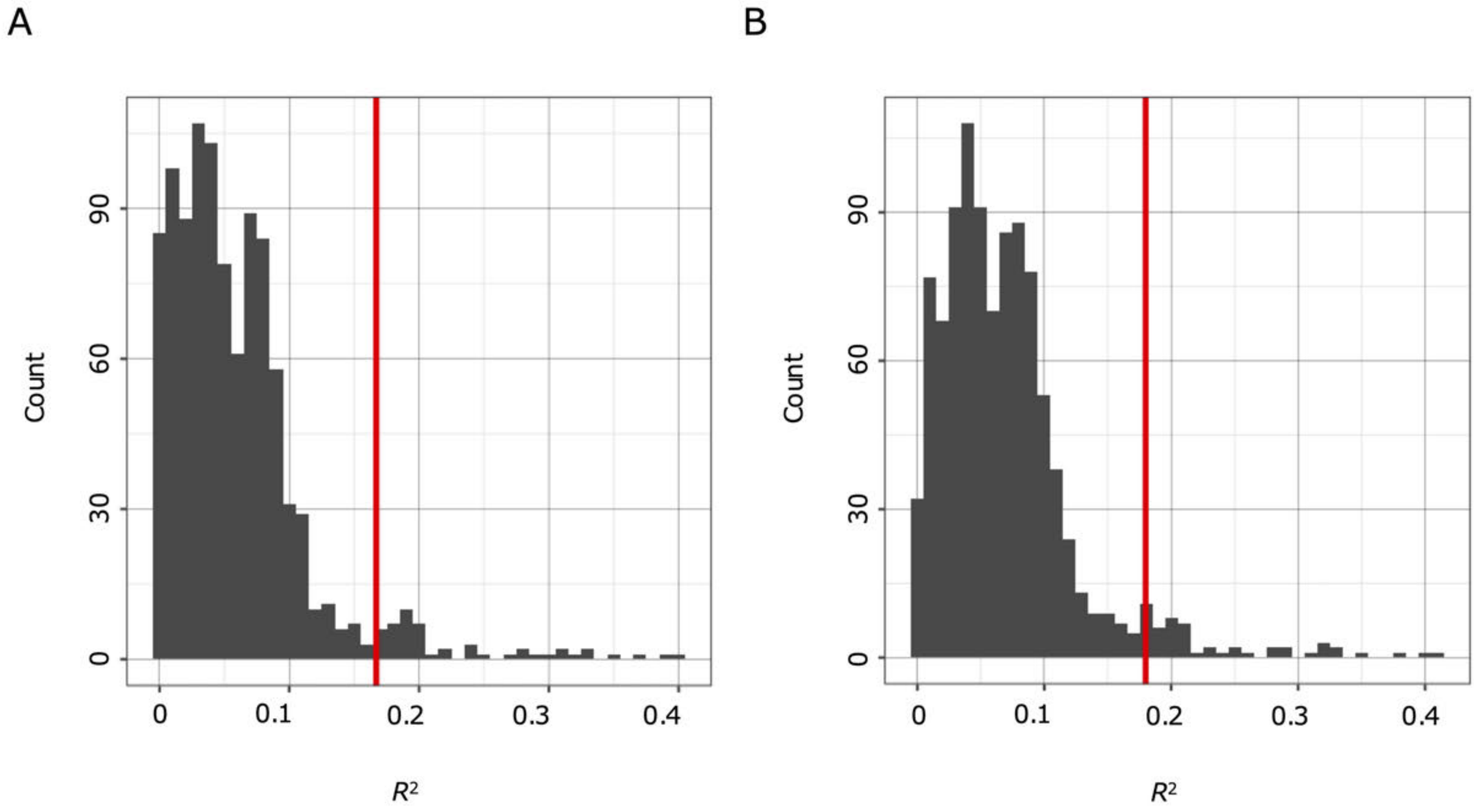

Supplementary Figure S2. Histograms of permutation of Model estimation
A and B show the histograms of coefficient of determinations (R^2) obtained by permutation tests for the 1st SNP and top two SNP combination, respectively. The red line indicates 5% for R^2.

A B

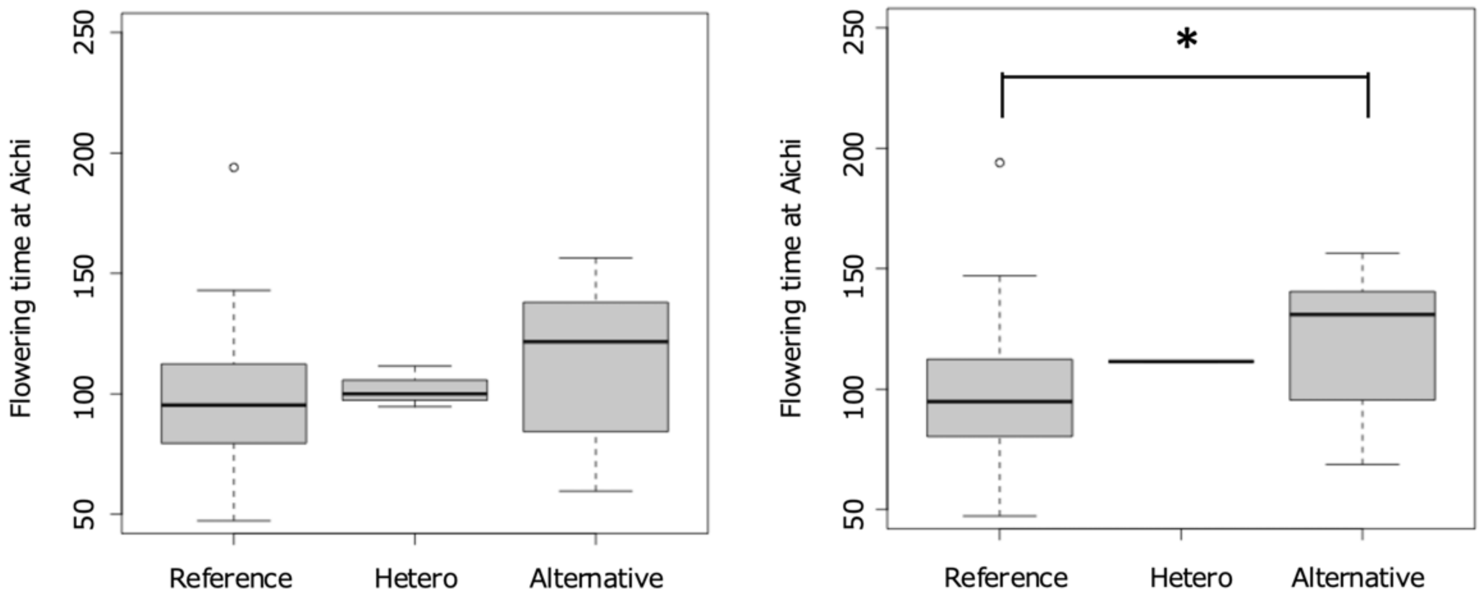

Supplementary Figure S3. Boxplot of flowering time for each genotype of the top 2 SNPs in subpopulation 3 shown in Shah et al. 2020

A and B show the boxplot of flowering time in Aichi for each genotype of 37 258 719 bp on chromosome 4 and 8 439 786 bp on chromosome 2, respectively, in subpopulation 3 shown in Shah et al. 2020. "Reference", "Alternative", and "hetero" respectively indicate homozygotes with the same genotype as the *L. japonicus* genome assembly build 3.0, homozygotes with a different genotype, and heterozygotes of both. Asterisks indicate $p < 0.05$.

Shah, N., Wakabayashi, T., Kawamura, Y., Skovbjerg, C.K., Wang, M.-Z., Mustamin, Y., et al. (2020) *Extreme genetic signatures of local adaptation during Lotus japonicus colonization of Japan*. *Nat Commun*, **11**.
